# The role of monocytes and macrophages in the progression of Alzheimer’s disease

**DOI:** 10.3389/fimmu.2025.1590909

**Published:** 2025-04-29

**Authors:** Wenyi Nie, Yingbin Yue, Jingqing Hu

**Affiliations:** ^1^ School of Traditional Chinese Medicine, Changchun University of Chinese Medicine, Changchun, China; ^2^ Department of Pediatrics, The First Affiliated Hospital of Xinjiang Medical University, Urumqi, China; ^3^ State Key Laboratory of Pathogenesis, Prevention and Treatment of High Incidence Diseases in Central Asia, Xinjiang Medical University, Urumqi, China; ^4^ School of Traditional Chinese Medicine, Tianjin University of Traditional Chinese Medicine, Tianjin, China; ^5^ Institute of Basic Theory for Chinese Medicine, China Academy of Chinese Medical Sciences, Beijing, China

**Keywords:** Alzheimer’s disease, monocytes, macrophages, neuroinflammation, β-Amyloid, MCP-1

## Abstract

Alzheimer’s disease (AD) is a progressive neurodegenerative disorder characterized by β-amyloid (Aβ) plaques, neurofibrillary tangles (NFTs), and neuroinflammation. Monocytes and macrophages, particularly microglia, play a dual role in AD pathogenesis. In the early stages, they delay disease progression by phagocytosing Aβ, but chronic activation leads to Aβ accumulation and exacerbated neuroinflammation. Monocyte chemoattractant protein 1 (MCP-1) is a key regulator in neuroinflammation, Aβ deposition, and tau pathology, making it a potential therapeutic target. Moreover, recent breakthroughs in fluid and imaging biomarkers and targeted immunomodulatory agents underscore the growing importance of early diagnostic and therapeutic interventions. This review explores the complex interplay between monocytes, macrophages, and AD pathology, highlighting their roles in neuroinflammation, Aβ metabolism, and tau phosphorylation. Understanding these mechanisms offers new insights into developing effective diagnostic biomarkers and therapeutic strategies for AD.

## 1 Introduction

Alzheimer’s disease (AD) is the most prevalent neurodegenerative disorder, marked by progressive cognitive decline and the accumulation of neuropathological hallmarks, including extracellular β-amyloid (Aβ) plaques, intracellular neurofibrillary tangles (NFTs) composed of hyperphosphorylated tau, and a sustained neuroinflammatory response (1). While Aβ and tau pathologies remain central to AD pathogenesis, mounting evidence implicates neuroinflammation as a key driver of disease progression, linking immune dysregulation to neuronal dysfunction and cognitive impairment (2).

Recent studies highlight the intricate and context-dependent roles of neuroinflammatory mechanisms in AD. Among the mediators of neuroinflammation, monocyte chemoattractant protein 1 (MCP-1) has emerged as a critical regulator, abundantly localized in Aβ plaques and associated with reactive microglia (3, 4). MCP-1 orchestrates monocyte recruitment to the brain, exacerbating neuroinflammation while also modulating Aβ clearance and tau hyperphosphorylation. Similarly, immune cells including microglia and infiltrating macrophages exhibit dual functions throughout disease progression. In early stages, microglia facilitate Aβ clearance, yet in chronic disease, they transition to a pro-inflammatory phenotype that perpetuates neuronal damage. Conversely, macrophages exhibit a complex interplay between Aβ/tau phagocytosis and the secretion of inflammatory cytokines, contributing to both protective and pathological outcomes (5, 6). These dynamic interactions underscore the necessity of delineating the precise mechanisms by which immune-inflammatory networks influence AD pathogenesis.

This review synthesizes recent advances in understanding MCP-1 and immune cells as pivotal mediators of AD pathology. We first examine the mechanisms by which MCP-1 bridges neuroinflammation with Aβ and tau aggregation. Further, we dissect the evolving roles of microglia and macrophages across distinct disease stages, emphasizing their dual neuroprotective and neurotoxic effects. Finally, we explore emerging therapeutic strategies targeting neuroinflammatory pathways, offering perspectives on potential interventions to mitigate AD progression.

## 2 Evolving paradigms in AD pathogenesis

### 2.1 Aβ dysregulation from homeostasis to pathology

The etiology of AD remains unclear, and its pathogenesis is extremely complex, primarily characterized by the abnormal deposition of Aβ in the brain and the presence of neurofibrillary tangles composed mainly of Tau protein. The mainstream theories of AD pathogenesis are the Aβ hypothesis and the Tau protein hypothesis, although other hypotheses have also emerged. Aβ is a metabolic product derived from the cleavage of amyloid precursor protein (APP) by β-secretase and γ-secretase. Under physiological conditions, Aβ is non-toxic, and its production and clearance are in dynamic equilibrium. However, in AD patients, increased generation or reduced clearance of Aβ leads to its abnormal deposition in the brain, forming insoluble amyloid plaques. These plaques activate microglia, triggering inflammatory responses that ultimately result in neuronal death and cognitive decline (2, 7). Research indicates that the formation of Aβ plaques is closely associated with abnormal processing of APP (8). APP is cleaved by β-secretase and γ-secretase to produce insoluble Aβ peptides, and post-translational modifications such as pyroglutamation and phosphorylation of Aβ reduce its degradation, leading to Aβ accumulation (9). The gradual accumulation of Aβ plaques impairs normal neuronal function and spreads to other brain regions, further exacerbating neuronal damage (10).

### 2.2 Tau hyperphosphorylation and neuronal collapse

Tau protein is a crucial component of neuronal microtubules, participating in microtubule assembly and stability, and maintaining cytoskeletal integrity. In AD patients, tau protein undergoes hyperphosphorylation, losing its normal microtubule-binding capacity and aggregating into paired helical filaments and straight filaments, ultimately forming NFTs. The presence of NFTs disrupts neuronal structure and function, leading to synaptic loss, neuronal death, and cognitive decline (11). The hyperphosphorylation of tau protein is closely associated with the activation of cyclin-dependent kinase 5 (CDK5). Aβ activates calpain, which regulates p35, the primary activator of CDK5, causing its cleavage into p25 under high-calcium conditions, leading to excessive activation of CDK5. The overactivation of CDK5 further promotes the hyperphosphorylation of tau protein, forming NFTs and contributing to the neurodegenerative cascade in AD (12). Emerging biomarker p-Tau181 is linked to tau pathology, and neurofilament light chain (NfL) reflect axonal damage, when combined enhance diagnostic accuracy and provide a comprehensive understanding of AD pathology (13).

### 2.3 Neuroinflammation as a driver of AD progression

Neuroinflammation is a significant feature in the pathogenesis of AD. The presence of Aβ plaques and NFTs activates microglia, leading to the release of pro-inflammatory cytokines and chemokines (14), further exacerbating neuroinflammation and neuronal damage. Microglia, the resident macrophages in the CNS, are responsible for maintaining neuronal homeostasis. In the early stages of AD, microglia delay disease progression by phagocytosing Aβ. However, under chronic inflammatory conditions, the persistent activation of microglia leads to Aβ accumulation and exacerbation of neuroinflammation (15). The presence of Aβ plaques and NFTs causes synaptic damage and increased reactive oxidative stress, acting as pathological triggers that induce the sustained activation of Toll-like receptors 2 (TLR2), TLR4, TLR6, and their co-receptors on microglia, upregulating the secretion of pro-inflammatory cytokines and chemokines (16). The activation of cyclin-dependent kinases further promotes tau hyperphosphorylation and increases Aβ plaque formation, thereby worsening cognitive dysfunction in AD patients (17). In addition to the Aβ hypothesis, tau hypothesis, and neuroinflammation hypothesis, recent years have seen the proposal of viral, mitochondrial dysfunction, insulin signaling abnormalities, gut microbiota dysbiosis, excitatory amino acid toxicity, and cholinergic dysfunction hypotheses, further enriching the research on the pathogenesis of AD (6, 18–22). The pathogenesis of AD is extremely complex, with many influencing factors. A single hypothesis is difficult to explain the occurrence and development of AD. This also shows that its specific mechanism is still unclear and requires more basic research for in-depth exploration.

## 3 The role of MCP-1 in AD

### 3.1 MCP-1 origins, signaling pathways, and neuroinflammatory cascades

Abnormal levels of Aβ and tau protein phosphorylation in the brains of AD patients lead to plaque formation and NFTs, further activating microglia and inducing neuroinflammation, thereby promoting disease progression. Studies have found a significant correlation between MCP-1 gene polymorphisms and AD risk, and MCP-1 plays a crucial regulatory role in neuroinflammation, Aβ deposition, and tau phosphorylation (**Table 1**). MCP-1, also known as CC chemokine ligand 2 (CCL2), is primarily derived from epithelial cells, smooth muscle cells, fibroblasts, endothelial cells, mononuclear macrophages, astrocytes, and microglia. Its primary receptor is CC chemokine receptor 2 (CCR2). In the brain, MCP-1 is primarily produced by astrocytes and resident microglia, with a smaller contribution from endothelial cells. This chemokine-receptor interaction plays a critical role in mediating immune cell recruitment and inflammatory responses, highlighting its significance in various physiological and pathological processes. MCP-1 binds to CCR2, activating downstream signaling pathways and regulating the chemotaxis of monocytes, thereby participating in the development and progression of various diseases (23). In AD patients, the expression of MCP-1 is significantly elevated, particularly around senile plaques and in reactive microglia, indicating its important role in the neuroinflammatory and pathological processes of AD (5).

**Table 1:** MCP-1 in alzheimer’s disease: mechanisms and therapeutic implications.

Pathological Process	Mechanisms	Signaling Pathways
Neuroinflammation	Recruit monocytes/macrophages; activates microglia to release IL-1β, TNF-α, ROS.	CCR2/PI3K-AKT, JAK-STAT
Aβ Deposition	Upregulates ApoE expression; disrupts BBB to enhance monocyte infiltration.	CCR2-mediated chemotaxis
Tau Hyperphosphorylation	Induces phosphatase dysregulation; activates microglia to promote tau aggregation.	CDK5/p25 pathway
Therapeutic Targets	Blocking CCR2 signaling; modulating ApoE expression; enhancing BBB integrity.	Smad2/3, NLRP3 inhibition

Through binding to CCR2, MCP-1 triggers downstream signaling cascades, including the phosphatidylinositol 3-kinase/protein kinase B (PI3K/AKT) pathway and the Janus kinase/signal transducer and activator of transcription (JAK/STAT) pathway, both of which regulate immune cell infiltration and inflammation-related gene expression (16). In AD, these signaling events are closely tied to the disease’s neuroinflammatory landscape. Neuroinflammation is a central feature of AD pathogenesis, and MCP-1 directly contributes to its progression by recruiting monocytes that differentiate into macrophages within the CNS parenchyma, reinforcing the pro-inflammatory milieu (24). Elevated MCP-1 and its downstream mediators accumulate near senile plaques, promoting both local immune activation and further Aβ-driven inflammatory responses (25). Consequently, a feedforward cycle emerges in which MCP-1’s origins and receptor-mediated signaling drive robust neuroinflammatory cascades. The sustained infiltration of peripheral immune cells, coupled with microglial activation, ultimately fosters the chronic inflammatory state characteristic of AD. This persistent inflammation not only impairs Aβ clearance but also accelerates tau hyperphosphorylation, together exacerbating neuronal damage and cognitive decline.

### 3.2 MCP-1 in Aβ dynamics from clearance to accumulation

MCP-1 plays a significant role in Aβ deposition through several mechanisms, including the upregulation of apolipoprotein E (ApoE) expression, the accumulation of monocyte-derived macrophages in the brain, and a mutually reinforcing relationship with Aβ deposition. ApoE is the primary apolipoprotein in the CNS, mainly produced by astrocytes and microglia, and plays a crucial role in promoting the hydrolysis and clearance of Aβ. Research indicates that MCP-1 can enhance the expression of ApoE, thereby influencing Aβ deposition. In AD transgenic mouse models, the complete loss of ApoE significantly reduces Aβ deposition, while the upregulation of MCP-1 increases Aβ plaque formation (26). Additionally, MCP-1 disrupts the blood-brain barrier (BBB), promoting the migration and infiltration of peripheral monocytes into the brain. In the context of AD, sustained elevation of MCP-1 levels leads to BBB disruption, significantly increasing the infiltration of monocyte-derived macrophages and exacerbating Aβ deposition (27). The role of MCP-1 in tau pathology is gradually being uncovered. Studies have shown that MCP-1 upregulates phosphatase activity, inducing the hyperphosphorylation of tau protein and promoting the formation of NFTs. In mouse models of neuroinflammatory diseases, the overexpression of MCP-1 significantly increases the accumulation and phosphorylation levels of tau protein (28). Furthermore, MCP-1 activates microglia, promoting the spread and aggregation of tau protein, thereby exacerbating the pathological processes of AD.

## 4 Macrophage heterogeneity and functional dynamics in AD

### 4.1 Diverse macrophage subsets in the CNS

Macrophages, especially CNS-resident microglia (derived from monocytes), are crucial immune regulators with dual roles in AD pathogenesis (29). They influence AD progression via Aβ phagocytosis, neuroinflammation, and tau pathology exacerbation (30, 31). Early in AD, microglia clear soluble Aβ via TREM2/CD36, while peripheral macrophages aid Aβ removal (32, 33). Chronic Aβ exposure, however, shifts microglia toward a pro-inflammatory state (NLRP3 activation, IL-1β/TNF-α release), worsening synaptic dysfunction and neurodegeneration (34). Microglia also drive tau hyperphosphorylation (via GSK-3β/CDK5) and spread tau via exosomes (35), highlighting their shift from protective to detrimental roles. Macrophages within the CNS are stratified into parenchymal and non-parenchymal subsets (36). Parenchymal macrophages are exclusively represented by microglia, which originate from embryonic yolk sac progenitors and sustain neuronal homeostasis via synaptic refinement and metabolic support. Non-parenchymal populations include perivascular macrophages (PVM) localized at the BBB interface and monocyte-derived macrophages recruited during neuroinflammatory states (37, 38). Under physiological conditions, microglia dynamically patrol the CNS through ramified processes, while PVM regulate BBB permeability and participate in clearance of interstitial waste. In AD pathogenesis, both subsets initially engage in Aβ phagocytosis via TREM2-dependent mechanisms, but persistent Aβ exposure drives their transition toward a pro-inflammatory phenotype, exacerbating tau pathology and synaptic loss (39) (**Table 2**).

**Table 2:** Macrophage subsets and their functional dynamics in alzheimer’s disease.

Macrophage Subset	Origin and Localization	Early stage	Late stage	Molecules/Pathways
Microglia	CNS-resident, yolk sac-derived	Phagocytosis of Aβ oligomers, protective inflammation	Prolonged activation, neurotoxic inflammation, synaptic dysfunction, tau hyperphosphorylation	TREM2, CD36, NLRP3 inflammasome, CDK5, GSK-3β
Peripheral Monocyte-Derived Macrophages	Derived from circulating monocytes, infiltrate CNS via choroid plexus, meninges, and compromised BBB regions	Aβ phagocytosis, secretion of Aβ-degrading enzymes (neprilysin, IDE)	Phenotypic switch, reduced phagocytic efficiency, sustained neuroinflammation, metabolic dysfunction	TREM2, CD36, NLRP3 inflammasome, LRP1, MMP-9, VCAM-1
Perivascular Macrophages (PVM)	Located in perivascular spaces adjacent to cerebral blood vessels	Regulation of BBB permeability, clearance of vascular Aβ	Reduced clearance of Aβ, increased vascular amyloid burden, role in cerebral amyloid angiopathy	BBB integrity, ROS production

### 4.2 Microglia in AD shifting from protection to pathology

Microglia are unique tissue-resident macrophages (TRMs) in the CNS, functioning through self-renewal. Under homeostatic conditions, microglia remain in a resting state, but upon detecting threats to the CNS, they rapidly transition to an activated state, migrating to sites of injury to phagocytose pathogens (40). Microglial activation can be beneficial or detrimental, depending on disease stage and microenvironment. In early/moderate AD, some subsets promote Aβ clearance and suppress neurotoxic inflammation (41), whereas in advanced stages, chronic Aβ exposure and cytokine dysregulation drive a pro-inflammatory phenotype that exacerbates synaptic damage and neurodegeneration (42, 43). Single-cell studies reveal distinct microglial subsets with transcriptional profiles linked to protective or harmful roles (44–46), underscoring their context-dependent functions in AD. These divergent findings highlight ongoing debates, emphasizing that timing, location, and cellular milieu determine whether microglia aid or worsen AD progression. Inflammatory factors play vital roles in diseases progression (47–50). Molecular pathways driving this phenotypic shift include persistent TLR4/TREM2 signaling, which sustains inflammation, and NLRP3 inflammasome activation, promoting IL-1β/IL-18 release (51). Dysregulated calcium homeostasis and elevated ROS activate kinases like GSK-3β, amplifying pro-inflammatory genes while reducing Aβ-clearing receptors (52), collectively shifting microglia toward a neurotoxic state.

Microglia also drive AD-associated neuroinflammation. Aβ-activated microglia release pro-inflammatory cytokines, including interleukin-1β (IL-1β), tumor necrosis factor-α (TNF-α), and interleukin-6 (IL-6), as well as ROS and nitric oxide (NO) (53). While initially protective, chronic activation perpetuates synaptic dysfunction, neuronal loss, and Aβ deposition, creating a pathological feedback loop (54). Aβ-induced inflammasome upregulation (e.g., NLRP3) further amplifies inflammation and neuronal damage. Additionally, tau can activate microglia, and excessive Aβ stimulation induces immune dysfunction, triggering inflammatory responses (55). Activated microglia release neurotoxic mediators, damaging healthy neurons and promoting hyperphosphorylated tau production. This cycle impairs synaptic integrity, reduces excitatory potentials, and drives neurodegeneration, culminating in cognitive decline, motor deficits, and behavioral abnormalities characteristic of AD (56).

### 4.3 Peripheral monocyte-derived macrophages in Aβ clearance

Non-parenchymal macrophages, often termed peripheral-derived macrophages, constitute a ubiquitous immune population differentiated from circulating monocytes that transmigrate across vascular endothelia. These cells exhibit remarkable plasticity, adapting to tissue-specific microenvironments through epigenetic reprogramming and metabolic shifts (e.g., oxidative phosphorylation to glycolysis). In AD, monocyte-derived macrophages infiltrate the CNS via three primary routes: choroid plexus stromal channels (regulated by CCL2-CCR2 chemotaxis), leptomeningeal vasculature (guided by VCAM-1 integrin signaling), and compromised BBB regions (mediated by MMP-9-dependent endothelial remodeling). During early AD, these macrophages exert neuroprotective effects through TREM2/CD36-dependent phagocytosis of soluble Aβ species and secretion of Aβ-degrading enzymes (neprilysin, IDE). However, sustained exposure to Aβ oligomers induces a phenotypic switch characterized by phagocytic receptor downregulation (e.g., LRP1), lysosomal acidification failure, and NLRP3 inflammasome-driven IL-1β/IL-18 hypersecretion. This functional decline coincides with Aβ plaque maturation and microglial priming, creating a feedforward loop of neuroinflammation. Notably, single-cell transcriptomics reveals that AD-associated macrophages adopt a disease-associated metabolic profile (DAMP), marked by lipid droplet accumulation and impaired mitophagy, which correlates with their diminished Aβ clearance capacity in late-stage patients (57). Postmortem studies further confirm that while CD14^+^CD16^+^ monocyte-derived macrophages accumulate near cerebral amyloid angiopathy sites, their Aβ-binding receptors (SCARA1, RAGE) are internalized and degraded, rendering them functionally inert—a critical factor driving clinical disease progression (58).

### 4.4 Perivascular macrophages and amyloid clearance

PVM are a unique type of brain macrophage, closely associated with the cerebral vascular system, located in the perivascular spaces of cerebral resistance arteries. PVM play roles in brain infection, immune activation, AD, and vascular-cognitive dysfunction through interactions with brain endothelial cells, circulating macrophages, and the production of reactive oxygen species. Studies in AD mouse models have shown that a reduction in PVM significantly increases vascular Aβ levels, and modulating PVM density can affect the clearance of amyloid from cerebral vessels. Conversely, stimulating PVM turnover can reduce cerebral amyloid angiopathy burden independently of microglia clearance, highlighting the importance of PVM in AD (59). In summary, existing evidence supports that PVM are a crucial component of the brain-resident immune system and play a protective role in the pathogenesis of AD.

### 4.5 Therapeutic strategies targeting macrophage plasticity

Progression of AD and targeting macrophage mechanisms may represent a promising therapeutic strategy for AD, aiming to shift cellular phenotypes from neurotoxic to neuroprotective states. Animal studies have demonstrated that osteopontin (OPN) can accelerate the recruitment of monocyte-derived macrophages to the brains of AD mice, promoting macrophage polarization toward an anti-inflammatory, highly phagocytic phenotype. This process inhibits Aβ production and aggregation while enhancing Aβ clearance, thereby potentially preventing AD progression (60). Additionally, blocking the Smad2/3 signaling pathway has been shown to effectively reduce Aβ levels in peripheral organs and circulation, enhance Aβ efflux from the brain, and improve Aβ clearance by peripheral macrophages. These effects collectively reduce Aβ deposition, neuroinflammation, and cognitive deficits in the brain (61). These findings highlight the therapeutic potential of modulating macrophage function and signaling pathways to mitigate AD pathology. In this context, targeting MCP-1 represents a particularly compelling avenue for therapeutic intervention (62). Experimental approaches to reduce MCP-1 or block its receptor CCR2 have shown promise in dampening neuroinflammation and modulating macrophage phenotypes (63). By inhibiting the MCP-1/CCR2 axis, it may be possible to decrease the infiltration of pro-inflammatory monocytes into the brain and simultaneously promote a reparative or Aβ-clearing macrophage phenotype (63, 64), these findings underscore the potential for translating MCP-1 modulation into tangible AD treatments.

## 5 Conclusion

Monocytes and macrophages play a crucial role in the pathogenesis of AD. MCP-1 regulates the migration and infiltration of monocytes, participating in neuroinflammation, Aβ deposition, and tau pathology. Macrophages, particularly microglia, delay disease progression by phagocytosing Aβ in the early stages of AD, but their persistent activation under chronic inflammatory conditions leads to Aβ accumulation and exacerbation of neuroinflammation. Future research should further explore the specific molecular mechanisms of MCP-1 and macrophages in AD, particularly their interactions in neuroinflammation, Aβ metabolism, and tau pathology. Based on the regulatory mechanisms of MCP-1 and macrophages, the development of new therapeutic strategies and early diagnostic biomarkers holds promise for advancing the prevention and treatment of AD. Recent advances in single-cell RNA sequencing, spatial transcriptomics, and advanced imaging enable high-resolution spatiotemporal analysis of MCP-1 and monocyte-derived cells in Alzheimer’s disease, revealing their dynamic roles in neuroinflammation and offering insights for stage-specific interventions. These technologies map immune cell heterogeneity, migration, and activation, linking molecular profiles to AD pathology like Aβ plaques and tau deposits (13). In summary, monocytes and macrophages occupy a central position in the complex pathological network of AD, and future research should continue to explore their multifaceted roles, with the aim of developing more effective diagnostic and therapeutic strategies, offering new hope for AD patients.
